# Impact of 19 years of mass drug administration with ivermectin on epilepsy burden in a hyperendemic onchocerciasis area in Cameroon

**DOI:** 10.1186/s13071-019-3345-7

**Published:** 2019-03-19

**Authors:** Charlotte Boullé, Alfred K. Njamnshi, Fidèle Dema, Michel K. Mengnjo, Joseph Nelson Siewe Fodjo, Anne-Cécile Zoung-Kanyi Bissek, Patrick Suykerbuyk, Cédric G. Lenou-Nanga, Hugues C. Nana-Djeunga, Joseph Kamgno, Cédric B. Chesnais, Michel Boussinesq, Robert Colebunders

**Affiliations:** 1 TransVIHMI, University of Montpellier, Inserm, IRD, Montpellier, France; 2 Infectious and Tropical Diseases Department, University Hospital, Nîmes, France; 3 Neurology Department, Central Hospital of Yaoundé, Yaoundé, Cameroon; 4 Faculty of Medicine and Biomedical Sciences, University of Yaoundé I, Yaoundé, Cameroon; 5 Brain Research Africa Initiative (BRAIN), Yaoundé, Cameroon; 6 District Hospital of Yoko, Yoko, Cameroon; 7 Subdivisonal hospital of Mbangassina, Mbangassina, Cameroon; 8 Global Health Institute, University of Antwerp, Antwerp, Belgium; 9 Ministry of Public Health, Yaoundé, Cameroon; 10 Dermatology Department, Chantal Biya Mother-Child Center, Yaoundé, Cameroon; 11 Centre for Research on Filariasis and other Tropical Diseases, Yaoundé, Cameroon

**Keywords:** Cameroon, Epilepsy, Onchocerciasis, Community study

## Abstract

**Background:**

Surveys conducted in 1991–1992 in the Mbam Valley (Cameroon) revealed that onchocerciasis was highly endemic, with community microfilarial loads (CMFL) > 100 microfilariae/snip in some villages. Also in 1991–1992, a survey of suspected cases of epilepsy (SCE) found 746 SCE using a questionnaire administered to individuals identified by key informants, with prevalences reaching 13.6% in some communities. From 1998, annual community-directed treatment with ivermectin (CDTI) was implemented to control onchocerciasis. In 2017, a door-to-door household survey was conducted in three of the villages visited in 1991–1992, using a standardized 5-item epilepsy screening questionnaire.

**Results:**

In 2017, a total of 2286 individuals living in 324 households were screened (582 in Bayomen, 553 in Ngongol and 1151 in Nyamongo) and 112 SCE were identified (4.9%). Neurologists examined 92 of these SCE and confirmed the diagnosis of epilepsy for 81 of them (3.5%). Between the surveys in 1991–1992 and 2017, the prevalence of SCE decreased from 13.6% to 2.5% in Bayomen (*P* = 0.001), from 8.7% to 6.6% in Ngongol (*P* = 0.205) and from 6.4% to 5.4% in Nyamongo (*P* = 0.282). The median age of SCE shifted from 20 (IQR: 12–23) to 29 years (IQR: 18–33; *P* = 0.018) in Bayomen, from 16 (IQR: 12–21) to 26 years (IQR: 21–39; *P* < 0.001) in Ngongol and from 16 (IQR: 13–19) to 24 years (IQR: 19–32; *P* < 0.001) in Nyamongo. The proportions of SCE aged < 10, 10–19, 20–29 and ≥ 30 years shifted from 9.5, 58.3, 25.0 and 7.1% in 1991–1992 to 2.7, 20.5, 39.3 and 37.5% in 2017, respectively.

**Conclusions:**

SCE prevalence decreased overall between 1991–1992 and 2017. The age shift observed is probably due to a decrease in the number of new cases of epilepsy resulting from the dramatic reduction of *Onchocerca volvulus* transmission after 19 years of CDTI.

**Electronic supplementary material:**

The online version of this article (10.1186/s13071-019-3345-7) contains supplementary material, which is available to authorized users.

## Background

In 2010 it was estimated that the number of people in the world with lifetime epilepsy (i.e. with a history of epilepsy, regardless of treatment or recent seizure activity) and with active epilepsy (i.e. who has had at least one epileptic seizure in the previous 5 years, regardless of antiepileptic drug treatment) were 68.8 and 32.7 million, respectively [1]. However, important geographical disparities exist and an estimated 80% of people living with epilepsy (PWE) reside in low and middle-income countries [1, 2]. Moreover, estimated prevalences in Africa are amongst the highest observed worldwide, with 0.9% and 6.0% in sub-Saharan Africa and central Africa, respectively [3]. Incidence rates of epilepsy in sub-Saharan Africa range from 64 to 187 per 100,000 person-years [4]. Evident causes including perinatal brain damage or parasitic diseases that are highly endemic in sub-Saharan Africa partly account for the high epilepsy burden endured by the region. Indeed, recent meta-analyses confirmed that neurocysticercosis, toxocariasis, toxoplasmosis and cerebral malaria are closely associated with epilepsy, with common odds ratios of 2.7 (95% confidence interval, CI: 2.1–3.6), 1.69 (95% CI: 1.42–2.01), 2.25 (95% CI: 1.27–3.9) and 4.68 (95% CI: 2.52–5.70), respectively [5–8].

*Onchocerca volvulus*, the nematode causing onchocerciasis that is transmitted from human to human by blackflies of the genus *Simulium*, has also been associated with epilepsy as early as 1938 [9]. More recent studies and meta-analyses have confirmed that this association is significant (pooled odds ratio: 2.49, 95% CI: 1.60–3.86) [10–14], though the causal relationship and the pathological mechanisms involved are yet to be demonstrated and elucidated.

During the past 25 years, all African onchocerciasis-endemic countries have established national control programmes based on annual mass community-directed treatment with ivermectin (CDTI). Recent observations in the Democratic Republic of Congo (DRC) and northern Uganda suggest that onchocerciasis control through CDTI (plus vector control in the case of Uganda) may decrease the incidence of epilepsy [15–17]. The benefit of CDTI may result from the primary prevention of new cases of *O. volvulus* infection or the reduction in microfilarial density during childhood, even though an improvement in the frequency or severity of seizures after ivermectin treatment has also been suggested [18].

This study aimed at assessing the impact of repeated CDTI campaigns on (i) epilepsy prevalence and (ii) age distribution of suspected cases of epilepsy (SCE), in three villages of the Mbam Valley, Cameroon. These villages are located in an onchocerciasis-endemic area that we initially surveyed in 1991–1992, and in 2017 after a 25-year interval, including 19 years of CDTI implementation.

## Methods

### Study sites and procedures for the 1991–1992 surveys

Parasitological surveys were conducted in 25 villages of the Mbam Valley (“Mbam et Kim” and “Mbam et Inoubou” divisions, Centre Region, Cameroon) between 1991 and 1992, to measure the levels of infection with *O. volvulus* [19]. In view of the concerns about epilepsy raised by the local population, 14 villages were selected for assessing the number of SCE. The local population had a fairly good knowledge of the condition [20], including the two main types of epilepsy: generalized tonic-clonic seizures (“persons who fall”) and absence seizures. For each village, after sensitization of the community leaders, the village chiefs gathered at one location all those who were seen in the village as having epilepsy. Where a subject did not wish to come to the meeting point, they were visited at home. After recording his/her full name, sex and age, each subject was questioned separately by a physician and a nurse, together with close relatives who had witnessed one or more of the epileptic episodes. Individuals were considered to be suspected of having epilepsy if one of the following situations occurred at least twice without obvious cause: (i) fell to the ground with loss of consciousness; (ii) had shaking movements of the arms or legs without control; or (iii) seemed to be absent and did not answer questions asked by relatives. Careful questioning on the frequency and the circumstances of the seizures, the age at first seizure, and the anti-epileptic drugs taken allowed to distinguish SCE from other conditions such as isolated seizures or febrile convulsions. In most cases, SCE presented with typical symptoms of generalized tonic-clonic seizures or absence seizures. SCE were not re-examined by a neurologist to confirm the diagnosis of epilepsy. To calculate prevalences, the total number of people living in each village was extrapolated from the second general population and housing census of 1987, for which data by village was available, and a growth rate of 2.3% per year was applied.

### Study sites and procedures for the 2017 survey

**Fig. 1:**
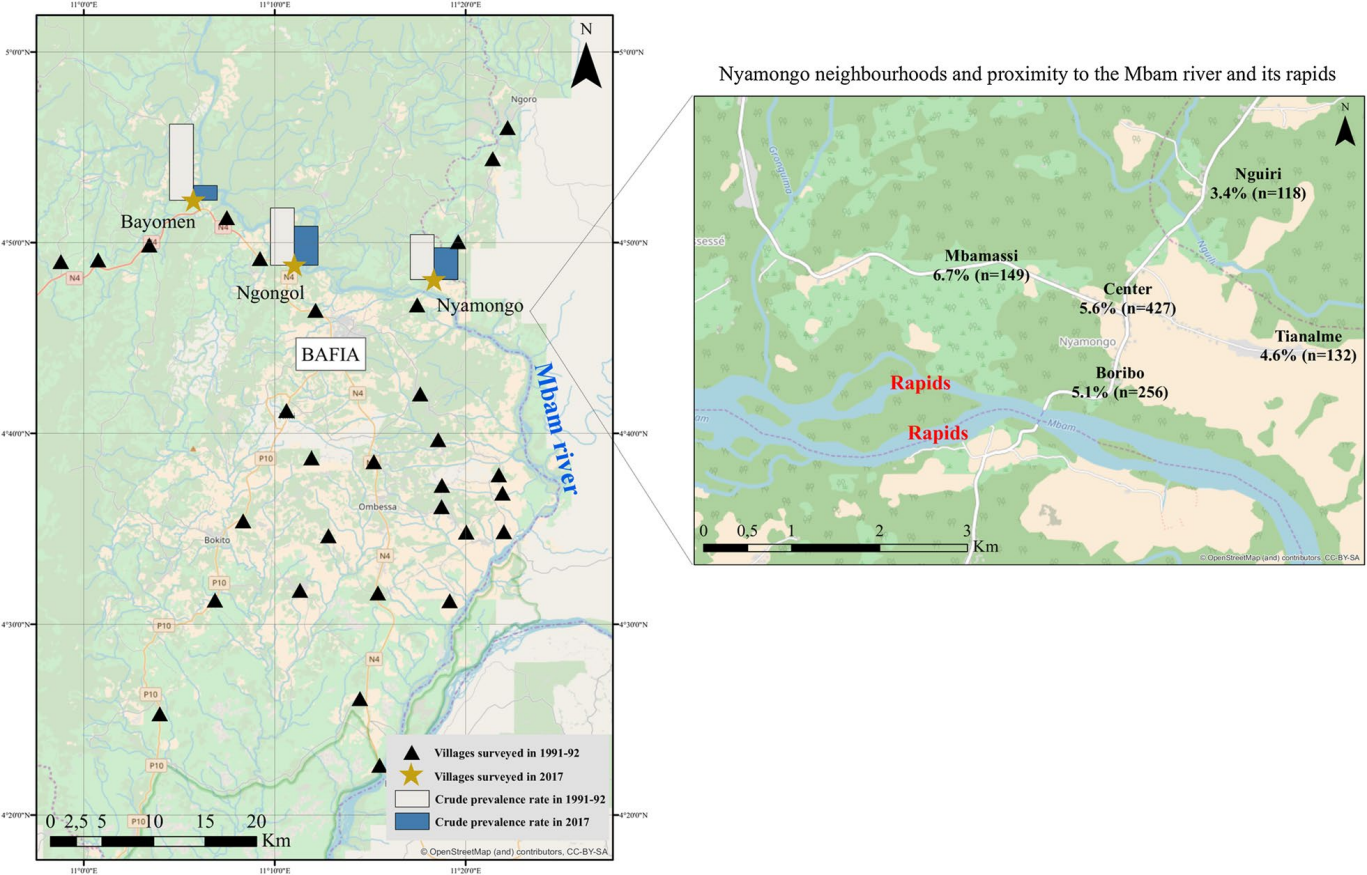
Map of the study area. Crude prevalence estimates are based on the suspected cases of epilepsy. The map was created CBC by using ArcGIS v.10.3.1. (ESRI 2018). The basemap layer was retrieved from OpenStreetMap and contributors, CC-BY-SA (http://www.openstreetmap.org)

Three of the 14 communities surveyed in 1991–1992 were selected to be revisited in 2017, namely Bayomen (initial proportion of SCE: 13.6%, 15/110), Nyamongo (6.4%, 40/627) and Ngongol (8.7%, 28/323). The selection was done using two criteria: (i) the initial (1991–1992) prevalence of SCE had to be high to ensure sufficient statistical power while comparing to 2017 data; and (ii) they had to be fairly well separated from neighboring communities to prevent confusion during the population census in 2017. These villages are located in an area of a forest-savanna mosaic, 100 km north of Yaoundé (Cameroon’s capital city), and less than 2 km from the Mbam river with its numerous rapids suitable for *Simulium* breeding (Fig. 1). In 1991–1992, the community microfilarial load (CMFL, defined as the geometric mean of the individual microfilarial densities per skin snip in those aged ≥ 20 years, calculated after having added 1 to each density to include zero counts), was 88.4 microfilariae per skin snip (mf/ss) in Ngongol and 46.8 mf/ss in Nyamongo. The CMFL was not measured in Bayomen, but was certainly also very high. The principal activity of the people in this region is agriculture and to a lesser extent fishing and sand mining in the river. From 1998 onwards, all villages in the study area benefitted from annual CDTI developed by the African Programme for Onchocerciasis Control (APOC) but no vector control activity was ever conducted in the area. Additionally, the study area appears to be either free or subjected to a marginal impact of other epileptogenic parasitic diseases (i.e. cysticercosis, paragonimiasis, trypanosomiasis) [21–23].

A door-to-door household epilepsy survey was conducted in the three selected villages in July 2017. A community questionnaire was administered to the key informants (village chiefs and secretaries) to obtain information on the village and its evolution over the last 25 years. The community health workers and/or ivermectin distributors who were trusted by the local population guided the investigators throughout the village and served as interpreters when necessary. Investigators provided adequate information about the study and obtained written consent from the household head before collecting demographic information (age and sex) for all the household members, and administering a validated 5-item questionnaire for epilepsy screening (see Additional file 1) [24]. The items were: (i) history of loss of consciousness and/or loss of bladder control and/or drooling; (ii) history of absence or sudden lapse of consciousness during a short period; (iii) history of sudden unintentional clonic movements or muscular jerks of arms and/or legs (convulsions) that stopped within minutes; (iv) history of sudden and brief bodily sensations, seeing or hearing things that are not there, or smelling strange odors; and (v) having been told that he/she had epilepsy or had experienced 2 epileptic episodes. SCE were asked whether they had already taken ivermectin and examined for the presence of cutaneous signs of onchocerciasis. Data related to the place of birth and the occupation of the household head, as well as the history of death due to epilepsy within the household, was also collected from the household head. Indigenous households were defined as those in which the household head was born in the village.

### Case definitions

People who screened positive for one or more questions in the 5-item questionnaire (including those taking anti-epileptic medication) were recorded as SCE and evaluated by a neurologist using a more detailed questionnaire. A SCE was considered as a PWE if there was a history of at least two unprovoked seizures separated by 24 h or more, therefore excluding seizures related to acute events such as fever, trauma, substance abuse or withdrawal [25]. The possible epilepsy (PE) group included PWE and SCE who could not be examined by the study neurologists, and therefore for whom diagnosis of epilepsy was not ruled out.

### Serologic assessment of the transmission of *O. volvulus* in 2017

Ongoing onchocerciasis transmission in the villages was assessed in children aged 7–10 years using the SD Bioline Onchocerciasis Ov16 IgG4 rapid diagnostic test (RDT) (Standard Diagnostics, Gyeonggido, South Korea) for anti-*Onchocerca* antibodies.

### Statistical analyses

Categorical variables were summarized using percentages and compared using Chi-square tests. Continuous variables were summarized using median and interquartile ranges (IQR) and compared using the non-parametric Wilcoxon-Mann-Whitney test.

Potential predictors for epilepsy were investigated using bivariate mixed model analysis. All variables with a *P*-value of < 0.20 in the bivariate analysis were entered into a multivariable logistic mixed model analysis with a backward approach. Multivariable models systematically included gender, village, and age (≤ 15, 15–30, > 30 years) as independent variables. A random effect was set at the household level and allowed for evaluation of the intra-class correlation coefficient. Adjusted odds ratios (aORs) were calculated with 95% confidence intervals (95% CI). Crude incidence rates were approached by using the number of SCE with onset of epilepsy in 1986–1990 or 2012–2016 (*n*) divided by the estimated number of people at risk during the same 5-year period (*N*). A 5-year period was used due to the small number of new events occurring in a 1-year time period. Assuming that the population in the villages was relatively stable during the two 5-year periods, we assumed that the populations at the end of these periods were that estimated for 1991 (from data of the 1987 national population census), and that recorded in 2017 (during the survey), respectively. The number of people with onset of epilepsy prior to 1986 or 2012 and the number of people for whom the year of onset of epilepsy was not known were subtracted from these populations to obtain the denominators on which the incidence rates were calculated. We considered that the death and migrations of new cases of epilepsy during the 5-year period would have a minimal effect on the incidence. The incidence of epilepsy was presented as cases per 100,000 person-years (PY). The significance level was set at *P* < 0.05. All *P*-values were two-sided. All analyses were performed using R software, v.3.4.2, and Stata v.13.1 software (StataCorp, College Station, USA).

### Standardization method

Crude prevalences were compared using Chi-square tests. Standardized prevalences were calculated using the sex and age structure of the population in the villages (age groups: 0–4, 5–9, 10–14, 15–19, 20–29, 30–39, > 40 years). In the 1991–1992 survey, the numbers of individuals in a given category were estimated using the total population in the village and the age and sex structure of the rural population recorded during the 1987 national census in the districts where the study villages are located. In order to enable a comparison of the prevalences between the surveys in 1991–1992 and 2017, a direct standardization was then performed by weighting the estimated SCE prevalences by sex and age group of 1991 using the age and sex structure of the population surveyed in 2017 [26].

### Sensitivity analysis

The primary analyses were conducted on the total number of PWE identified in 2017. Sensitivity analysis was performed on the SCE and the PE cases. In addition, the survey conducted in 2017 revealed that significant immigration occurred into these villages between the surveys in 1991–1992 and 2017 (see “Results”). The CMFL in 1991 in the study villages were extremely high (see above), and subjects who arrived in these communities between the two surveys most probably came from areas with lower exposure to *O. volvulus*, although we lack information on their exact origin. Thus, this immigration might have led to an artificial reduction in the CMFL in the villages and, should high microfilarial densities cause epilepsy, to an artificial reduction in the SCE prevalence in 2017. To prevent the risk of an over-detection of a decrease in prevalence between 1991 and 2017, we conducted a sensitivity analysis restricted to the members of the indigenous households.

## Results

### Study population

A total of 324 households were screened in 2017, consisting of 2286 individuals: 582 (88 households) in Bayomen; 553 (86 households) in Ngongol; and 1151 (150 households) in Nyamongo. Individuals’ and household characteristics are presented in Table 1. There were 1148 women (50.2%) in the study population and the median age was 18 years (interquartile range, IQR: 7–33).

**Table 1:** Characteristics of individuals and households surveyed in 2017. Indigenous households were defined as those in which the household head was born in the village

Characteristic	Total	Bayomen	Ngongol	Nyamongo
Individuals’ characteristics	*n* = 2286	*n* = 582	*n* = 553	*n* = 1151
Age (years), median (IQR)	18 (7–33)	15 (6–32)	20 (8–35)	18 (8–33)
Female, *n* (%)	1148 (50.2)	306 (52.6)	261 (47.2)	581 (50.5)
Male, *n* (%)	1138 (49.8)	276 (47.2)	292 (52.8)	570 (49.5)
Household characteristics	*n* = 324	*n* = 88	*n* = 86	*n* = 150
Number of people per household, median (IQR)	6 (4–9)	6 (4–9)	6 (4–8)	7 (5–10)
Number of households whose head’s occupation is agriculture, *n* (%)	278 (85.8)	70 (79.6)	73 (84.9)	135 (90.0)
Number of indigenous households, *n* (%)	173 (53.4)	25 (28.4)	35 (40.7)	113 (75.3)
For migrants, year of immigration, median (IQR)	2008 (1997–2014)^a^	2012 (2002–2015)	2002 (1987–2013)^a^	2003 (1992–2013)^a^
Number of households with				
1 SCE, *n* (%)	59 (18.2)	12 (13.6)	23 (26.7)	24 (16.0)
2 SCE, *n* (%)	14 (4.3)	2 (2.3)	4 (4.7)	8 (5.3)
3 or more SCE, *n* (%)	7 (2.2)	1 (1.1)	1 (1.2)	5 (3.3)
Number of households with history of death from epilepsy, *n* (%)	52 (16.1)^a^	12 (13.6)	16 (18.6)^a^	24 (16.0)^a^
Year of death, median (IQR)	2007 (2000–2011)	2005 (1999–2009)	2009 (1999–2013)	2007 (2001–2010)
Age at death (years), median (IQR)	20 (15–27)	19 (14–28)	21 (15–27)	20 (17–29)
Number of SCE, PWE and PE				
In the whole population				
SCE, *n* (%)	112 (4.9)	19 (3.3)	34 (6.2)	59 (5.1)
PWE, *n* (%)	81 (3.5)	15 (2.6)	24 (4.3)	42 (3.7)
PE, *n* (%)	101 (4.4)	17 (2.9)	29 (5.2)	55 (4.8)
In the indigenous population				
SCE, *n* (%)	79 (5.9)	14 (6.7)	15 (6.5)	50 (5.5)
PWE, *n* (%)	58 (4.3)	11 (5.3)	10 (4.3)	37 (4.1)
PE, *n* (%)	71 (5.3)	13 (6.3)	12 (5.2)	46 (5.1)
Characteristics of SCE, PWE and PE				
Age, median (IQR)				
SCE	26 (20–34)	29 (18–33)	26 (21–39)	24 (19–32)
PWE	24 (20–32)	26 (18–33)	24 (20–31)	24 (20–32)
PE	24 (20–32)	29 (21–33)	23 (20–28)	24 (19–30)
Not epilepsy patient	46 (20–70)			
Age at onset (years), median (IQR)^b^	12 (10–14)^a^	11 (9–13)	11 (9–13)	13 (10–15)^a^
Year of onset, median (IQR)^b^	2004 (1996–2010)^a^	2002 (1995–2011)	2003 (1994–2009)	2006 (1996–2010)^a^
Type of seizures				
Only NS, *n* (%)^b^	2 (2.5)	0	0	2 (4.8)
NS and other types of seizures, *n* (%)^b^	21 (25.9)	3 (20.0)	9 (37.5)	9 (21.4)
Other types of seizures, *n* (%)^b^	58 (71.6)	12 (80.0)	15 (62.5)	31 (73.8)

^a^Data missing

^b^For PWE only

*Abbreviations*: SCE, suspected case of epilepsy; PWE, persons living with epilepsy; PE, persons with possible epilepsy (PWE and SCE who could not be examined by a neurologist); NS, nodding seizures

The proportion of indigenous households varied from 28.4% in Bayomen to 40.7% in Ngongol and 75.3% in Nyamongo. The proportion of households that had moved in from other locations was particularly high in Bayomen where the median year of arrival of the individuals recorded in 2017 was 2012 (IQR: 2002–2015). The median age of the indigenous and non-indigenous residents was 18 (IQR: 7–35) and 17 years (IQR: 7–32) (*P* = 0.059), respectively. Agriculture was the main declared occupation for 85.8% (*n* = 278) of the household heads, and each household housed a median of 6 people (IQR: 4–9).

### Village data

In all three villages, CDTI was first implemented in 1998. In Bayomen, two people including the village chief and a certified nurse handled the drug distribution, with no interruption since 1998 (last distribution in June 2017). There was only one community distributor in Ngongol who reported no interruption in CDTI in the last 15 years (last distribution in July 2017), and two distributors in Nyamongo where the supply of ivermectin was repeatedly insufficient to ensure a good population coverage (last distribution in June 2016). There were no health centers in Bayomen and Ngongol. Antiepileptic drugs (AED) (phenobarbital and carbamazepine) were available in a private drug store in Bayomen, whereas in the other villages inhabitants had to buy AED in the city of Bafia (see Fig. 1). In Bayomen, the key informant reported a massive arrival of inhabitants who moved in from other regions of Cameroon, whereas there were only a few departures from Ngongol since 2015 without arrivals and no major change in Nyamongo.

### Suspected cases of epilepsy

In 2017, the 5-item questionnaire administered to the household heads (and the subjects if they were present) allowed the identification of 112 SCE (prevalence of SCE: 4.9%). Among those who were later confirmed to be PWE, 71 (87.7%) had a positive answer to question 1, 33 (40.7%) to question 2, 65 (80.3%) to question 3, 10 (12.4%) to question 4 and 72 (88.9%) to question 5 [“have you (he/she) been told that you (he/she) had epilepsy or had had 2 epileptic episodes?”]. In contrast, only 3 of the 11 SCE (27.3%) for whom the diagnosis of epilepsy was refuted had a positive answer to this question 5. Fifty-two households (16.1%) had already lost one member, likely due to epilepsy (suspected), at a median age of 20 years (IQR: 15–27) (Table 1).

### Confirmed cases of active epilepsy

**Fig. 2:**
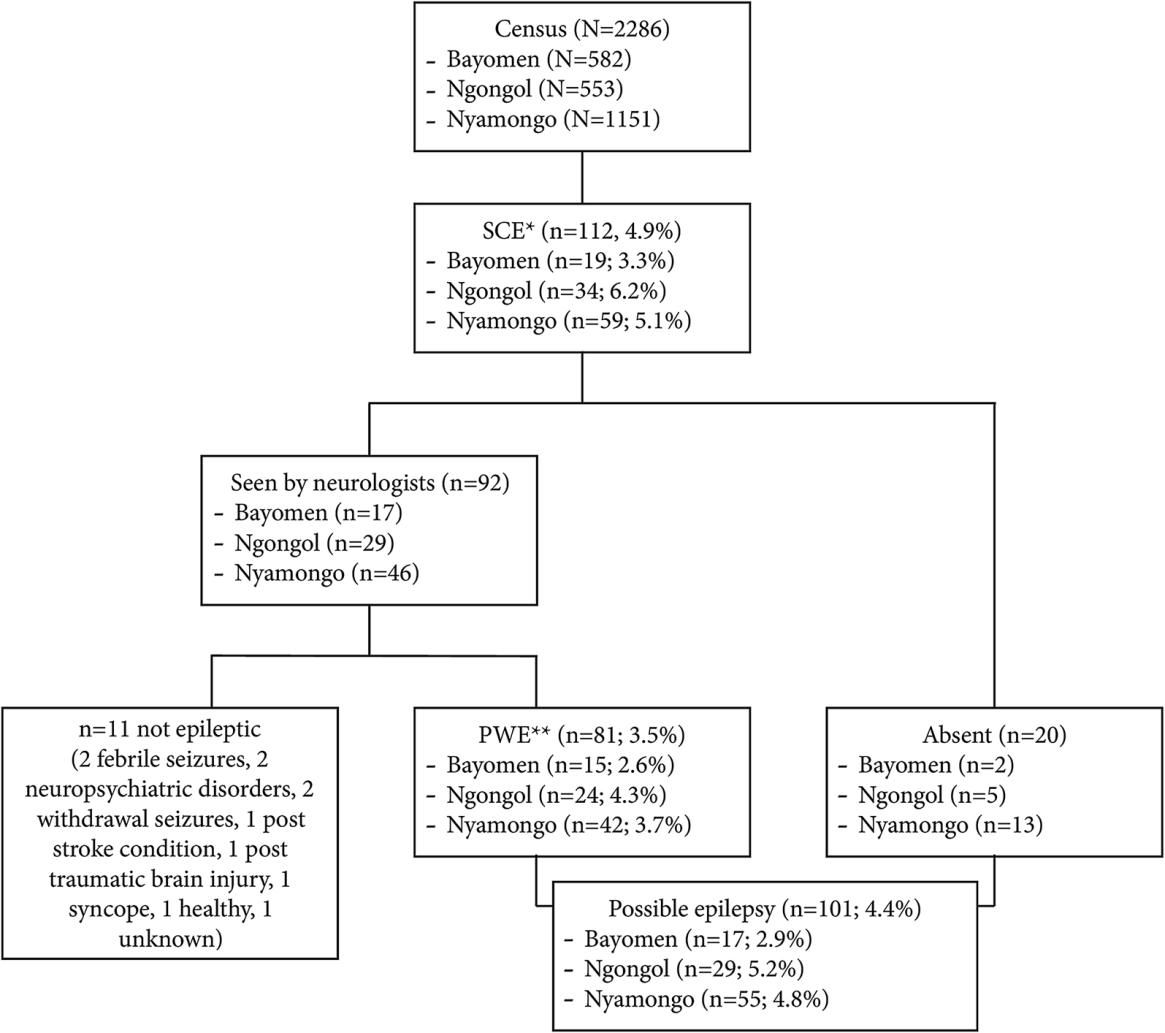
Study flowchart. *SCE, suspected case of epilepsy; **PWE, person with epilepsy

In 2017, 92 of the 112 SCE could be examined by neurologists, who confirmed the diagnosis of active epilepsy in 81 of them (positive predictive value: 88.0%) (Fig. 2). Therefore, the prevalence of confirmed epilepsy was 3.5%. Twenty SCE could not be examined by neurologists, mainly because they were absent during the survey (i.e. the questionnaire was administered only to the household head), raising the prevalence of possible epilepsy to 4.4% (*n* = 101). The median age of unexamined SCE was 25 years (IQR: 20–30), 11 (55%) were males, and 16 (80%) had provided a positive answer to question 5.

In univariate analysis, factors associated with the PWE status were age, living in an indigenous household, and a history of death due to epilepsy within the household (Table 2). In multivariable mixed model analysis, and using the individuals aged < 15 years as the reference group, age remained strongly associated with PWE status, with aOR = 11.6 (95% CI: 5.4–25.4; *P* < 0.001) for the 15–30 age group and aOR = 3.9 (95% CI: 1.7–9.0; *P* = 0.002) for the > 30 age group. Indigenous households remained associated with a higher risk (aOR = 2.1; 95% CI: 1.1–4.2; *P* = 0.026), but having a household history of death due to epilepsy was no longer significant. The results obtained as part of the sensitivity analysis were similar, whatever the populations of epilepsy cases considered (SCE, PWE or PE cases) (Table 3). The random effect associated with the household was significant (*P* < 0.001), with an intra-class correlation coefficient equal to 23%.

**Table 2:** Factors associated with epileptic status in the univariate analyses

	PWE			SCE			Possible epilepsy		
	OR	95% CI	*P*	OR	95% CI	*P*	OR	95% CI	*P*
Male sex (ref: female)	1.03	0.65–1.63	0.893	1.00	0.67–1.48	0.990	1.06	0.70–1.61	0.766
Village									
Bayomen	ref			ref			ref		
Ngongol	1.95	0.90–4.25	0.091	2.18	1.10–4.30	0.025	2.12	1.03–4.37	0.042
Nyamongo	1.36	0.67–2.77	0.396	1.59	0.85–2.96	0.147	1.69	0.88–3.26	0.116
Age									
< 15 years	ref			ref			ref		
15–30 years	12.20	5.64–26.40	<0.0001	9.71	5.22–18.05	<0.0001	11.29	5.79–22.01	<0.0001
> 30 years	4.40	1.92–10.10	<0.0001	4.07	2.10–7.91	<0.0001	3.78	1.83–7.82	<0.0001
Lives in an indigenous household (ref: no)	1.78	1.00–3.18	0.049	1.71	1.05–2.81	0.032	1.69	1.01–2.84	0.046
Household history of death from epilepsy (ref: no)	1.93	1.00–3.70	0.050	2.30	1.33–3.96	0.003	1.83	1.01–3.30	0.047
Household head occupation: agriculture (ref: other)	1.98	0.72–5.44	0.184	1.32	0.61–2.84	0.481	1.73	0.72–4.13	0.218

**Table 3:** Factors associated with epileptic status in the multivariable mixed model analysis (*n* = 2246)

			*P*	aOR	95% CI	*P*	aOR	95% CI	*P*
Male sex (ref: female)	1.13	0.69–1.84	0.622	1.09	0.72–1.64	0.691	1.15	0.74–1.79	0.523
Village									
Bayomen	ref			ref			ref		
Ngongol	1.51	0.66–3.44	0.325	1.66	0.83–3.33	0.151	1.70	0.79–3.64	0.174
Nyamongo	0.85	0.39–1.87	0.688	1.09	0.56–2.10	0.802	1.18	0.58–2.43	0.647
Age									
< 15 years	ref			ref			ref		
15–30 years	11.64	5.35–25.36	<0.0001	9.01	4.84–16.15	<0.0001	10.70	5.46–20.96	<0.0001
> 30 years	3.85	1.66–8.98	0.002	3.53	1.80–6.92	<0.0001	3.37	1.61–7.05	0.001
Lives in an indigenous household (ref: no)	2.14	1.09–4.19	0.026	1.91	1.10–3.30	0.021	1.78	0.98–3.22	0.057
Household history of death from epilepsy (ref: no)	1.74	0.89–3.37	0.104	2.03	1.19–3.47	0.009	1.63	0.89–2.96	0.112

*Abbreviations*: PWE, person living with epilepsy; SCE, suspected case of epilepsy; OR, odds ratio; aOR, adjusted OR; ref, reference

### Comparison of the prevalences measured in 1991–1992 and 2017

**Fig. 3:**
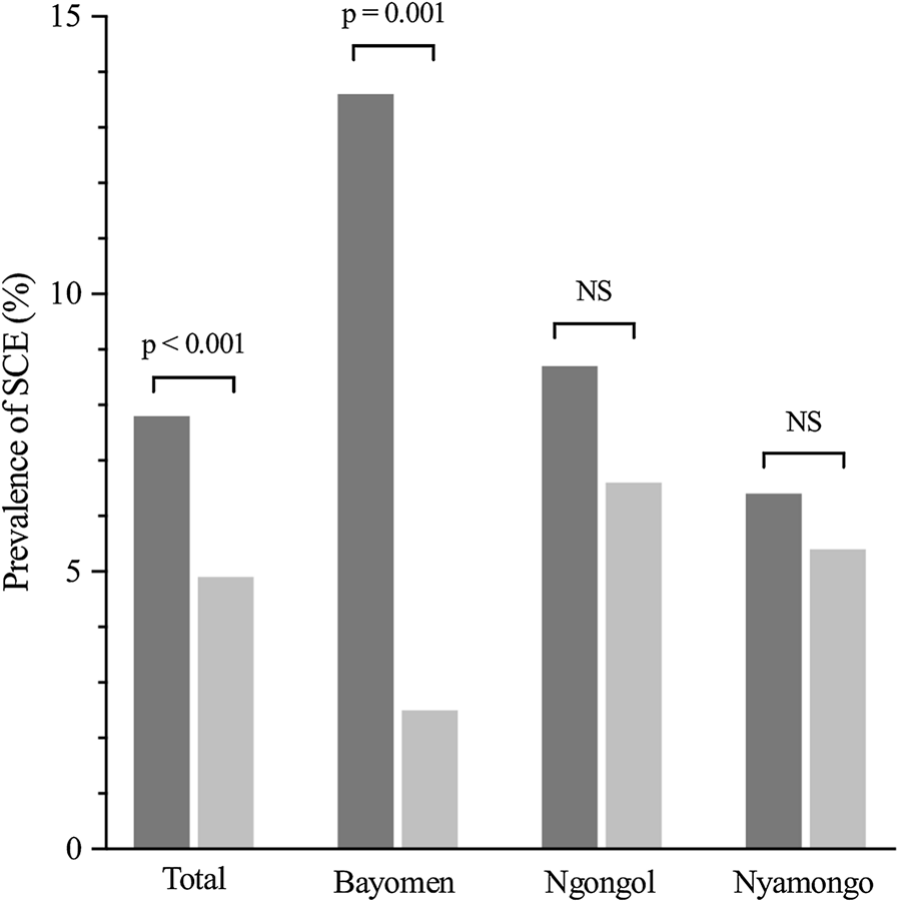
Variation in crude prevalences of SCE between 1991–1992 (dark grey) and 2017 (light grey). *Abbreviations*: SCE, suspected case of epilepsy; NS, no significant difference

The crude prevalence of SCE in the three villages decreased from 7.8% in 1991–1992 to 4.9% in 2017 (*P* < 0.001). When the villages were taken separately, the decrease was significant in Bayomen (from 13.6% to 3.3%; *P* = 0.001), but not in Ngongol (from 8.7% to 6.2%; *P* = 0.205) or in Nyamongo (from 6.4% to 5.1%; *P* = 0.282) (Fig. 3). When the analysis was restricted to indigenous households, the crude prevalences of SCE in 2017 were 6.7% (14/208) in Bayomen, 6.5% (15/232) in Ngongol and 5.5% (50/905) in Nyamongo. Considering that most of the SCE in 1991–1992 lived in indigenous households, the crude prevalence in this sub-population significantly decreased in Bayomen (*P* = 0.042) but not in Ngongol (*P* = 0.338) or Nyamongo (*P* = 0.484). When the SCE prevalence recorded in 1991–1992 was standardized on the age and sex structure of the population surveyed in 2017, standardized prevalences of 12.9% in Bayomen, 9.7% in Ngongol and 7.6% in Nyamongo were obtained. Comparisons of the prevalences standardized on the same sex- and age-structure show that they decreased significantly between the surveys in 1991–1992 and 2017 in the three villages (*P* < 0.001, *P* = 0.034 and *P* = 0.017, respectively).

### Shift in the age distribution of SCE between the surveys in 1991–1992 and 2017

**Fig. 4:**
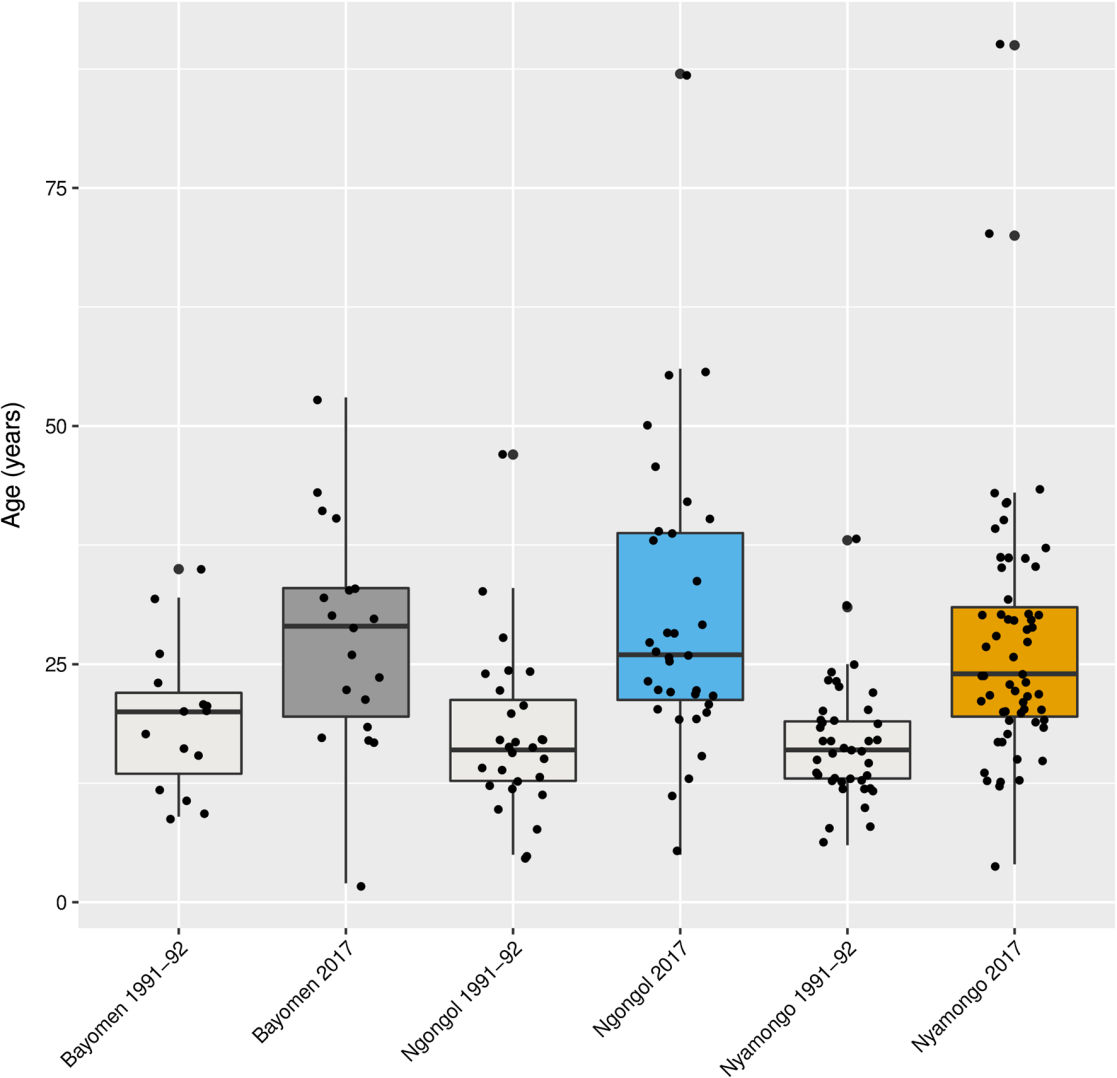
Age shift of the cohort of people living with epilepsy between 1991–1992 and 2017

Considering the three villages together, the median age of SCE increased from 16 years in 1991 (IQR: 13–21) to 26 years in 2017 (IQR: 20–34; *P* < 0.001) (Table 4). At the village level, SCE age increased from 20 (IQR: 12–23) to 29 years (IQR: 18–33; *P* = 0.018) in Bayomen, from 16 (IQR: 12–21) to 26 years (IQR: 21–39; *P* < 0.001) in Ngongol and from 16 (IQR: 13–19) to 24 years (IQR: 19–32; *P* < 0.001) in Nyamongo (Fig. 4). In 1991–1992, 49 (58.3%) of the SCE were 10–19 years-old (prevalence of 22.3%), whereas 21 (25.0%) were 20–29 years-old (prevalence of 16.3%). In 2017, only 23 (20.5%) of the SCE were 10–19 years-old (prevalence of 4.9%), whereas the majority (*n* = 44; 39.3%) were 20–29 years-old (prevalence of 11.6%) (Table 4).

**Table 4:** Comparison of the age characteristics of the epilepsy cases in 1991–1992 and 2017

	1991–1992	2017			
	SCE	SCE	*P*	PWE	*P*
Age (years), median (IQR)	16 (13–21)	26 (20–34)	<0.001	24 (20–32)	<0.001
Age group					
< 10 years, *n* (%)	8 (9.5)	3 (2.7)		1 (1.2)	
10–19 years, *n* (%)	49 (58.3)	23 (20.5)		18 (22.2)	
20–29 years, *n* (%)	21 (25.0)	44 (39.3)		34 (42.0)	
≥ 30 years, *n* (%)	6 (7.1)	42 (37.5)		28 (34.6)	
Crude age-specific prevalence^a^			<0.001		<0.001
< 10 years, (%)	2.5	0.4		0.1	
10–19 years, (%)	22.3	4.9		3.8	
20–29 years, (%)	16.3	11.6		9.0	
≥ 30 years, (%)	1.6	6.0		4.0	
Age at onset (years), median (IQR)	12 (8–15)^b^	na		12 (10–14)^c^	0.294
Age group at onset					
< 10 years, *n* (%)	21 (25.0)			19 (24.4)	
10–19 years, *n* (%)	49 (58.3)			57 (73.1)	
20–29 years, *n* (%)	5 (6.0)			1 (1.3)	
≥ 30 years, *n* (%)	1 (1.2)			1 (1.3)	
Years since onset of epilepsy, median (IQR)	5 (2.0–7.5)^b^	na		12.5 (7–21)^b^	<0.001

^a^Estimated number of people per age group (< 10, 10–19, 20–29 and ≥ 30 years) were obtained by applying the age structure of districts from the 1987 census

^b^8 missing data

*Abbreviation*: na, not available

The median age at first seizure of the PWE recorded in 2017 was 12 years (IQR: 10–14) and was similar in the three villages. This median age at first seizure was similar in the 1991–1992 survey (12 years, IQR: 8–15; *P* = 0.294). Those surveyed in 2017 had been living for a longer time with epilepsy (12.5 years, IQR: 7–21) as compared with 1991–1992 (5 years, IQR 2–7.5; *P* < 0.001).

### Incidence rates

Crude incidence rates were estimated to be roughly 802 SCE per 100,000 PY for the 1985–1990 period (*n* = 41 incident cases, *N* = 1060 at risk) as compared to 146 SCE per 100,000 PY for the 2012–2016 period (*n* = 16 incident cases including 2 in Bayomen, 4 in Ngongol and 10 in Nyamongo, among a total of 2190 at risk).

### Results of the Ov16 rapid diagnostic tests in children

A total of 307 children aged 7–10 years were examined (121 in Bayomen, 85 in Ngongol and 101 in Nyamongo). The proportions of Ov16-positive children did not differ significantly between the three villages (55.4, 42.4 and 46.5%, respectively; *P* = 0.156). The values tended to decrease with age (54.2, 51.9, 46.8 and 41.0% in children aged 7, 8, 9 and 10 years, respectively), the difference being not significant (*P* = 0.309).

## Discussion

Our study is, to our knowledge, the first study that has (i) permitted comparison of the burden of epilepsy in the same communities in a hyperendemic onchocerciasis area, before and after repeated rounds of CDTI (19 years since first implementation); (ii) used epilepsy screening tools that are very similar to those used before implementation of CDTI; and (iii) employed standardization techniques to ensure more accurate comparisons. Our study therefore brings new evidence to support the idea that CDTI has a significant impact on epilepsy burden.

Both crude and standardized epilepsy prevalences significantly decreased overall between the surveys in 1991–1992 and 2017. It could be argued that, with an estimation of 70% of survival at 10 years among people with epilepsy [27], and with the follow-up of 25 years in our study, more than half of the people living with epilepsy in 1991–1992 had died, therefore not contributing any more to the prevalences observed in 2017. Nevertheless, the prevalence of any endemic disease remains stable under the hypothesis that the incidence rate is counterbalanced by the mortality rate. The decrease in SCE prevalence that we observed can thus be either related to a rise in the mortality rate (which would imply worsening of the care of people living with epilepsy, seemingly unlikely given the trends towards gaining better access to medications and health facilities) or to a decrease in incidence. The age of onset of epilepsy remained unchanged between 1991–1992 and 2017. Meanwhile, PWE surveyed in 2017 were older, with a higher duration of disease, as compared with 1991–1992. Altogether these results imply a greater survival of PWE in 2017 and fewer new cases of epilepsy in recent years. It is therefore unlikely that there was a rise in mortality among PWE in our study population. Nevertheless, the incidence figures should be interpreted cautiously even though the crude rates show a drastic decrease after 25 years. Altogether our results highlight that the age shift in the PWE population can be interpreted as a cohort effect.

The difference in prevalence was striking in Bayomen, which was the only village where the decrease in the crude prevalence between 1991–1992 and 2017 was statistically significant. This difference between Bayomen and the two other villages might be due to (i) a higher CDTI coverage in Bayomen, where the village chief was also the ivermectin community distributor, whereas there were complaints in Ngongol and Nyamongo over shortages of ivermectin and suboptimal therapeutic coverage; (ii) an under- or overestimation of the SCE prevalences in 1991–1992; and/or (iii) the much higher immigration rate in Bayomen, leading to an artificial overestimation of the decrease in the prevalence in the village. Even if it is known that the sensitivity of the Ov16 RDT is lower than that of the Ov16 ELISA [28], the finding of a similar high prevalence of Ov16 antibodies in children 7–10 years-old in the three villages suggests a similar high degree of ongoing *O. volvulus* transmission, which could partly be due to programmatic reasons, including suboptimal ivermectin supply and poor monitoring. The observation that in 2017 the proportions of SCE and PWE in the total population of Bayomen (3.3 and 2.6%, respectively) were fairly low in comparison to those in the population of the indigenous households (6.7 and 5.3%, respectively) makes the latter hypothesis likely. The rapid increase in the size of Bayomen due to migration (population: 110 in 1991–1992 and 582 in 2017; to be compared with 323 and 553, respectively, in Ngongol; and to 627 and 1151, respectively, in Nyamongo) might also have led to a decrease in the individual exposure to blackflies (in the “vector-to-host ratio” [29]), and thus a slower increase in the *O. volvulus* microfilarial densities in the population, including the children.

Previous surveys conducted in onchocerciasis endemic areas in Africa before the introduction of CDTI showed that epilepsy prevalences were highest in the population aged 10–20 years (median age range 11–20 years; proportion in age group: 48.1–67.2% of all epileptic patients) [30–36]. Interestingly, two cross-sectional surveys conducted in Nigeria and Cameroon, in which several years of CDTI had been ongoing, showed that epilepsy prevalences were higher in the 20–29 years age group [37, 38]. These observations suggest that CDTI may reduce the incidence of epilepsy, although no baseline data were available. A case–control study conducted in onchocerciasis-endemic regions Democratic Republic of Congo (DRC) suggested that among people showing signs of onchocerciasis, those who took ivermectin were less likely to develop epilepsy than those who did not (OR = 0.52; 95% CI 0.28–0.98) [15]. To date, the exact mechanism driving the ascertained association between mass distribution of ivermectin and the decline in the prevalence of epilepsy remains to be elucidated. A recent study by our team showed a temporal relationship between onchocerciasis acquisition and development of epilepsy [39] suggesting that the action of ivermectin is likely mediated by its impact on *Onchocerca volvulus*, although it is also effective on other parasites. In studies performed before the introduction of ivermectin, microfilariae have been occasionally observed in cerebrospinal fluid [40, 41]. Therefore, the parasite itself may cause seizures or they may be caused by a more complex mechanism linked to a previous transit of parasites within the host immune system, including the hypothesis of the induction of a neurotoxic auto-immune response.

One limitation to our study resides in the lack of complete data on the present intensity of infection with *O. volvulus*, and on the therapeutic coverage achieved during the CDTI organized in the communities. However, the results of the examination of children by the Ov16 RDT demonstrate that transmission of onchocerciasis is ongoing at fairly high levels, thus corroborating the results of a parasitological survey conducted in 2015 in four villages of the study area, including Ngongol [42]. In the latter village, the prevalence of skin microfilariae decreased from 97.2% in 1992 to 57.0% in 2015 (a 41.4% reduction) but, more importantly, the CMFL decreased from 88.4 mf/ss to 1.6 mf/ss during the same period (98.2% reduction). This result supports the hypothesis that the impact of CDTI on the incidence of epilepsy is probably mainly due to a decrease in the quantitative indicators of transmission (CMFL and annual transmission potential in the vector population) and not in the skin microfilaria prevalence. This being said, a greater impact of CDTI on epilepsy would likely be achieved if drug coverage was higher in children. Unfortunately, another study conducted in the Bafia Health District showed that the coverage in 2014 was only 60% in children aged 5–9 years, and that the values increased progressively with age [43].

A second limitation of our study is that other etiologies of epilepsy could not be fully accounted for, and it could be suspected, for instance, that improved maternal care has reduced perinatal brain damage in newborns. Nevertheless, to our knowledge, no large-scale intervention against malaria is ongoing in the study area, other than the national bednet distribution programme. Notwithstanding these unaccounted factors, sensitivity analyses are reassuring on the consistency of our results, as data from the PWE or PE populations did not differ much from those from SCE.

Lastly, the definitions of suspect cases in 1991–1992 and 2017 were close, but not strictly identical. The main reason is that the first survey was conducted before a validated questionnaire for identifying the SCE was available. In 2017 all declared SCE cases were considered whereas some were excluded by questioning in 1991–1992, therefore marginally underestimating the initial SCE prevalences.

Interestingly, and independently from the principal objective of the study, we found a high positive predictive value of the 5-item questionnaire (88%) in our study area, that confirms its good screening accuracy [44]. Besides this, the analyses revealed the existence of a household effect, accounting for 23% of the variation in the risk of being a PWE. This can be explained by intrinsic genetic factors or by extrinsic factors shared by the household. The latter could be an intra-household similar intensity of exposure to blackfly bites, depending on the distance between the house or the workplace, and the river [45]. Figure 1 (right panel), suggests that in Nyamongo, like in other settings, there is a correlation between epilepsy and river proximity [19, 46].

## Conclusions

In conclusion, our study adds to the growing body of evidence for the impact of CDTI in decreasing the burden of epilepsy by limiting incident cases in hyperendemic onchocerciasis foci in Africa. Nevertheless, the still high epilepsy prevalence and the high prevalence of onchocerciasis antibodies in children 7–10 years-old, indicates a need for advocacy to strengthen onchocerciasis elimination programmes and improve comprehensive health services for PWE in these rural areas.

## Additional file

**Additional file 1.** Household questionnaire.

## Abbreviations

CDTI: Community-directed treatment with ivermectin
DRC: Democratic Republic of Congo
SCE: Suspected cases of epilepsy
PE: Possible epilepsy
PWE: Person with epilepsy
RDT: Rapid diagnostic rest
PY: Person-years
AED: Antiepileptic drugs

## References

[1] Ngugi AK, Bottomley C, Kleinschmidt I, Sander JW, Newton CR (2010). Estimation of the burden of active and life-time epilepsy: a meta-analytic approach. Epilepsia.

[2] Newton CR, García HH (2012). Epilepsy in poor regions of the world. Lancet.

[3] Ba-Diop A, Marin B, Druet-Cabanac M, Ngoungou EB, Newton CR, Preux PM (2014). Epidemiology, causes, and treatment of epilepsy in sub-Saharan Africa. Lancet Neurol.

[4] Ngugi AK, Kariuki SM, Bottomley C, Kleinschmidt I, Sander JW, Newton CR (2011). Incidence of epilepsy: a systematic review and meta-analysis. Neurology.

[5] Debacq G, Moyano LM, García HH, Boumediene F, Marin B, Ngoungou EB (2017). Systematic review and meta-analysis estimating association of cysticercosis and neurocysticercosis with epilepsy. PLoS Negl Trop Dis.

[6] Luna J, Cicero CE, Rateau G, Quattrocchi G, Marin B, Bruno E (2018). Updated evidence of the association between toxocariasis and epilepsy: systematic review and meta-analysis. PLoS Negl Trop Dis.

[7] Ngoungou EB, Bhalla D, Nzoghe A, Dardé ML, Preux PM (2015). Toxoplasmosis and epilepsy - systematic review and meta analysis. PLoS Negl Trop Dis.

[8] Christensen SS, Eslick GD (2015). Cerebral malaria as a risk factor for the development of epilepsy and other long-term neurological conditions: a meta-analysis. Trans R Soc Trop Med Hyg.

[9] Casis Sacre G (1938). El sindrome epileptico y su relacion con onchocercosis. Bol Salubr Hig.

[10] Druet-Cabanac M, Boussinesq M, Dongmo L, Farnarier G, Bouteille B, Preux PM (2004). Review of epidemiological studies searching for a relationship between onchocerciasis and epilepsy. Neuroepidemiology.

[11] Pion SDS, Kaiser C, Boutros-Toni F, Cournil A, Taylor MM, Meredith SEO (2009). Epilepsy in onchocerciasis endemic areas: systematic review and meta-analysis of population-based surveys. PLoS Negl Trop Dis.

[12] Kaiser C, Pion SDS, Boussinesq M (2013). Case-control studies on the relationship between onchocerciasis and epilepsy: systematic review and meta-analysis. PLoS Negl Trop Dis.

[13] Kamuyu G, Bottomley C, Mageto J, Lowe B, Wilkins PP, Noh JC (2014). Exposure to multiple parasites is associated with the prevalence of active convulsive epilepsy in sub-Saharan Africa. PLoS Negl Trop Dis.

[14] Ngugi AK, Bottomley C, Kleinschmidt I, Wagner RG, Kakooza-Mwesige A, Ae-Ngibise K (2013). Prevalence of active convulsive epilepsy in sub-Saharan Africa and associated risk factors: cross-sectional and case-control studies. Lancet Neurol.

[15] Levick B, Laudisoit A, Tepage F, Ensoy-Musoro C, Mandro M, Bonareri Osoro C (2017). High prevalence of epilepsy in onchocerciasis endemic regions in the Democratic Republic of the Congo. PLoS Negl Trop Dis.

[16] Colebunders R, Irani J, Post R (2016). Nodding syndrome - we can now prevent it. Int J Infect Dis.

[17] Spencer PS, Mazumder R, Palmer VS, Lasarev MR, Stadnik RC, King P (2016). Environmental, dietary and case-control study of Nodding Syndrome in Uganda: a post-measles brain disorder triggered by malnutrition?. J Neurol Sci.

[18] Kipp W, Burnham G, Kamugisha J (1992). Improvement in seizures after ivermectin. Lancet.

[19] Boussinesq M, Pion SDS, Demanga-Ngangue, Kamgno J (2002). Relationship between onchocerciasis and epilepsy: a matched case-control study in the Mbam Valley, Republic of Cameroon. Trans R Soc Trop Med Hyg.

[20] Njamnshi AK, Yepnjio FN, Bissek ACZK, Tabah EN, Ongolo-Zogo P, Dema F (2009). A survey of public knowledge, attitudes, and practices with respect to epilepsy in Badissa Village, Centre Region of Cameroon. Epilepsy Behav.

[21] Dongmo L, Druet-Cabanac M, Moyou SR, Zebaze DRM, Njamnshi AK, Sini V (2004). Cysticercosis and epilepsy: a case-control study in Mbam Valley, Cameroon. Bull Soc Pathol Exot.

[22] Ollivier G, Boussinesq M, Albaret JL, Cumberlidge N, Farhati K, Chippaux JP (1995). Epidemiological study of *Paragonimus* sp. in south Cameroon. Bull Soc Pathol Exot.

[23] Louis FJ, Louis-Lutinier D (1995). Le foyer historique de trypanosomiase de Bafia (Cameroun), objet de toutes les polémiques: revue bibliographique. Bull Liais Doc OCEAC.

[24] Preux PM (2000). Questionnaire in a study of epilepsy in tropical countries. Bull Soc Pathol Exot.

[25] Fisher RS, Acevedo C, Arzimanoglou A, Bogacz A, Cross JH, Elger CE (2014). ILAE official report: a practical clinical definition of epilepsy. Epilepsia.

[26] Minnesota Population Center. Integrated Public Use Microdata Series, International: Version 7.0 [dataset]. Minneapolis, MN, USA: IPUMS; 2018. 10.18128/D020.V70.

[27] Kamgno J, Pion SDS, Boussinesq M (2003). Demographic impact of epilepsy in Africa: results of a 10-year cohort study in a rural area of Cameroon. Epilepsia.

[28] World Health Organization. Report of the 1st meeting of the WHO onchocerciasis technical advisory subgroup, Varembé Conference Centre, Geneva, Switzerland, 10–12 October 2017. 2017. http://apps.who.int/iris/bitstream/handle/10665/273705/WHO-CDS-NTD-PCT-2018.05-eng.pdf. Accessed 4 Nov 2017.

[29] Remme JHF, Zongo JB, Service M (1989). Demographic aspects of the epidemiology and control of onchocerciasis in West Africa. Demography and vector-borne diseases.

[30] Kaiser C, Kipp W, Asaba G, Mugisa C, Kabagambe G (1996). The prevalence of epilepsy follows the distribution of onchocerciasis in a west Ugandan focus. Bull World Health Organ.

[31] Osuntokun BO (1978). Epidemiology of epilepsy in developing countries in Africa. Trop Geogr Med.

[32] Osuntokun BO, Adeuja AO, Nottidge VA, Bademosi O, Olumide A, Ige O (1987). Prevalence of the epilepsies in Nigerian Africans: a community-based study. Epilepsia.

[33] Diop AG, de Boer HM, Mandlhate C, Prilipko L, Meinardi H (2003). The global campaign against epilepsy in Africa. Acta Trop.

[34] Newell ED, Vyungimana F, Bradley JE (1997). Epilepsy, retarded growth and onchocerciasis, in two areas of different endemicity of onchocerciasis in Burundi. Trans R Soc Trop Med Hyg.

[35] König R, Nassri A, Meindl M, Matuja W, Kidunda AR, Siegmund V (2010). The role of *Onchocerca volvulus* in the development of epilepsy in a rural area of Tanzania. Parasitology.

[36] Ovuga E, Kipp W, Mungherera M, Kasoro S (1992). Epilepsy and retarded growth in a hyperendemic focus of onchocerciasis in rural western Uganda. East Afr Med J.

[37] Prischich F, De Rinaldis M, Bruno F, Egeo G, Santori C, Zappaterreno A (2008). High prevalence of epilepsy in a village in the Littoral Province of Cameroon. Epilepsy Res.

[38] Dozie INS, Onwuliri COE, Nwoke BEB, Chukwuocha UM, Chikwendu CI, Okoro I (2006). Onchocerciasis and epilepsy in parts of the Imo river basin, Nigeria: a preliminary report. Public Health.

[39] Chesnais CB, Nana-Djeunga HC, Njamnshi AK, Lenou-Nanga CG, Boullé C, Bissek AZ (2018). The temporal relationship between onchocerciasis and epilepsy: a population-based cohort study. Lancet Infect Dis.

[40] Mazzotti L (1959). Presencia de microfilarias de *Onchocerca volvulus* en el líquido cefaloraquídeo de enfermos tratados con hetrazan. Rev Inst Salubr Enferm Trop.

[41] Duke BOL, Vincelette J, Moore PJ (1976). Microfilariae in the cerebrospinal fluid, and neurological complications, during treatment of onchocerciasis with diethylcarbamazine. Tropenmed Parasitol.

[42] Kamga G-R, Dissak-Delon FN, Nana-Djeunga HC, Biholong BD, Mbigha-Ghogomu S, Souopgui J (2016). Still mesoendemic onchocerciasis in two Cameroonian community-directed treatment with ivermectin projects despite more than 15 years of mass treatment. Parasit Vectors.

[43] Kamga G-R, Dissak-Delon FN, Nana-Djeunga HC, Biholong BD, Ghogomu SM, Souopgui J (2018). Audit of the community-directed treatment with ivermectin (CDTI) for onchocerciasis and factors associated with adherence in three regions of Cameroon. Parasit Vectors.

[44] Diagana M, Preux PM, Tuillas M, Ould Hamady A, Druet-Cabanac M (2006). Dépistage de l’épilepsie en zones tropicales: validation d’un questionnaire en Mauritanie. Bull Soc Pathol Exot.

[45] Cadot E, Barbazan P, Boussinesq M (1998). Geographic determinants of onchocerciasis transmission in a forest-savannah transition zone: an example of 2 villages of the Mbam focus (central region, Cameroon). Sante.

[46] Colebunders R, Mandro M, Mokili JL, Mucinya G, Mambandu G, Pfarr K (2016). Risk factors for epilepsy in Bas-Uélé Province, Democratic Republic of the Congo: a case-control study. Int J Infect Dis.

