# Supplementary material for: Impact of 19 years of mass drug administration with ivermectin on epilepsy burden in a hyperendemic onchocerciasis area in Cameroon
Source: Parasit Vectors. 2019 Mar 19;12:114. doi: 10.1186/s13071-019-3345-7 (PMC6423875; doi:10.1186/s13071-019-3345-7)
Supplement: Supplementary file 1 — Additional file 1. Household questionnaire. [file 13071_2019_3345_MOESM1_ESM.pdf]

QUESTIONNAIRE AU NIVEAU MENAGE: DEPISTAGE de l'EPILEPSIE

Nom de l'enquêteur: ____ Signature:

Village: ___________________________ Date (JJ/MM/AAAA): __ __/__ __/201__

Caractéristiques du ménage:

CODE UNIQUE du ménage (E/VV/MMM): __ / __ __ /__ __ __

(E = numéro équipe; VV = numéro village; MMM = numéro ménage)

1) Nom du chef de ménage **(*)**: ___________________________________Tel

2) Est-ce que la famille est originaire du village: ☐ OUI ☐ NON Si non, depuis quand la famille vit dans le village? ____________ (années)

3) Activité principale génératrice de revenus de la famille:
☐ Agriculteur ☐ Eleveur de bétail ☐ Pêcheur ☐ Employé ☐ Artisan ☐ Autre, spécifiez: __________________________

4) Est-ce qu'un membre du ménage avec épilepsie est décédé? ☐ OUI ☐ NON ☐ NE SAIT PAS

Si OUI, Quand (année): ______________ A quel âge: _____________ ans
Si OUI, Quand (année): ______________ A quel âge: _____________ ans
Si OUI, Quand (année): ______________ A quel âge: _____________ ans
Si OUI, Quand (année): ______________ A quel âge: _____________ ans
Si OUI, Quand (année): ______________ A quel âge: _____________ ans

(*) Définition ménage: *l'ensemble des occupants d'un même logement sans que ces personnes soient nécessairement unies par des liens de parenté;*
L'INSEE utilise comme définition pour ménage le critère de consommation, c.-à-d. les personnes prenant leurs repas en commun.

DEPISTAGE EPILEPSIE: Pour chaque personne du ménage poser les 5 questions aux membres du ménage concerné
Si au moins 1 OUI, proposer de participer à l’étude et référer au neurologue. **[*IN : INconnu]**

**1. No. pers.**		**2. Age**	**3. Genre**		**4.1 QUESTION 1**			**4.2 QUESTION 2**			**4.3 QUESTION 3**			**4.4 QUESTION 4**			**4.5 QUESTION 5**		
Presént	Absent	(années ou mois) Exemple: 9 mois = 9M; 12 ans = 12A	M=masculin; F=féminin		Perte(s) de connaissance; avec ou sans perte d'urine; avec ou sans émission de bave ?			Absence(s) ou perte(s) de contact avec l'entourage de début brutal et de durée brève?			Secousses ou mouvements anormaux incontrôlables d'un ou des membres (convulsions), de début soudain et d’une durée de quelques minutes			Apparition brutale et de durée brève de sensations corporelles étranges, d’hallucinations ou d’illusions visuelles, auditives ou olfactives ?			A-t-on déjà dit au sujet qu’il était épileptique ou qu’il avait déjà fait **au moins 2 crises d’épilepsie ?**		
1 ☐	☐		M ☐	F ☐	OUI ☐	NON ☐	IN ☐	OUI ☐	NON ☐	IN ☐	OUI ☐	NON ☐	IN ☐	OUI ☐	NON ☐	IN ☐	OUI ☐	NON ☐	IN ☐
2 ☐	☐		M ☐	F ☐	OUI ☐	NON ☐	IN ☐	OUI ☐	NON ☐	IN ☐	OUI ☐	NON ☐	IN ☐	OUI ☐	NON ☐	IN ☐	OUI ☐	NON ☐	IN ☐
3 ☐	☐		M ☐	F ☐	OUI ☐	NON ☐	IN ☐	OUI ☐	NON ☐	IN ☐	OUI ☐	NON ☐	IN ☐	OUI ☐	NON ☐	IN ☐	OUI ☐	NON ☐	IN ☐
4 ☐	☐		M ☐	F ☐	OUI ☐	NON ☐	IN ☐	OUI ☐	NON ☐	IN ☐	OUI ☐	NON ☐	IN ☐	OUI ☐	NON ☐	IN ☐	OUI ☐	NON ☐	IN ☐
5 ☐	☐		M ☐	F ☐	OUI ☐	NON ☐	IN ☐	OUI ☐	NON ☐	IN ☐	OUI ☐	NON ☐	IN ☐	OUI ☐	NON ☐	IN ☐	OUI ☐	NON ☐	IN ☐
6 ☐	☐		M ☐	F ☐	OUI ☐	NON ☐	IN ☐	OUI ☐	NON ☐	IN ☐	OUI ☐	NON ☐	IN ☐	OUI ☐	NON ☐	IN ☐	OUI ☐	NON ☐	IN ☐
7 ☐	☐		M ☐	F ☐	OUI ☐	NON ☐	IN ☐	OUI ☐	NON ☐	IN ☐	OUI ☐	NON ☐	IN ☐	OUI ☐	NON ☐	IN ☐	OUI ☐	NON ☐	IN ☐
8 ☐	☐		M ☐	F ☐	OUI ☐	NON ☐	IN ☐	OUI ☐	NON ☐	IN ☐	OUI ☐	NON ☐	IN ☐	OUI ☐	NON ☐	IN ☐	OUI ☐	NON ☐	IN ☐
9 ☐	☐		M ☐	F ☐	OUI ☐	NON ☐	IN ☐	OUI ☐	NON ☐	IN ☐	OUI ☐	NON ☐	IN ☐	OUI ☐	NON ☐	IN ☐	OUI ☐	NON ☐	IN ☐
10 ☐	☐		M ☐	F ☐	OUI ☐	NON ☐	IN ☐	OUI ☐	NON ☐	IN ☐	OUI ☐	NON ☐	IN ☐	OUI ☐	NON ☐	IN ☐	OUI ☐	NON ☐	IN ☐
11 ☐	☐		M ☐	F ☐	OUI ☐	NON ☐	IN ☐	OUI ☐	NON ☐	IN ☐	OUI ☐	NON ☐	IN ☐	OUI ☐	NON ☐	IN ☐	OUI ☐	NON ☐	IN ☐
12 ☐	☐		M ☐	F ☐	OUI ☐	NON ☐	IN ☐	OUI ☐	NON ☐	IN ☐	OUI ☐	NON ☐	IN ☐	OUI ☐	NON ☐	IN ☐	OUI ☐	NON ☐	IN ☐
13 ☐	☐		M ☐	F ☐	OUI ☐	NON ☐	IN ☐	OUI ☐	NON ☐	IN ☐	OUI ☐	NON ☐	IN ☐	OUI ☐	NON ☐	IN ☐	OUI ☐	NON ☐	IN ☐
14 ☐	☐		M ☐	F ☐	OUI ☐	NON ☐	IN ☐	OUI ☐	NON ☐	IN ☐	OUI ☐	NON ☐	IN ☐	OUI ☐	NON ☐	IN ☐	OUI ☐	NON ☐	IN ☐
15 ☐	☐		M ☐	F ☐	OUI ☐	NON ☐	IN ☐	OUI ☐	NON ☐	IN ☐	OUI ☐	NON ☐	IN ☐	OUI ☐	NON ☐	IN ☐	OUI ☐	NON ☐	IN ☐
16 ☐	☐		M ☐	F ☐	OUI ☐	NON ☐	IN ☐	OUI ☐	NON ☐	IN ☐	OUI ☐	NON ☐	IN ☐	OUI ☐	NON ☐	IN ☐	OUI ☐	NON ☐	IN ☐
17 ☐	☐		M ☐	F ☐	OUI ☐	NON ☐	IN ☐	OUI ☐	NON ☐	IN ☐	OUI ☐	NON ☐	IN ☐	OUI ☐	NON ☐	IN ☐	OUI ☐	NON ☐	IN ☐
18 ☐	☐		M ☐	F ☐	OUI ☐	NON ☐	IN ☐	OUI ☐	NON ☐	IN ☐	OUI ☐	NON ☐	IN ☐	OUI ☐	NON ☐	IN ☐	OUI ☐	NON ☐	IN ☐
19 ☐	☐		M ☐	F ☐	OUI ☐	NON ☐	IN ☐	OUI ☐	NON ☐	IN ☐	OUI ☐	NON ☐	IN ☐	OUI ☐	NON ☐	IN ☐	OUI ☐	NON ☐	IN ☐
20 ☐	☐		M ☐	F ☐	OUI ☐	NON ☐	IN ☐	OUI ☐	NON ☐	IN ☐	OUI ☐	NON ☐	IN ☐	OUI ☐	NON ☐	IN ☐	OUI ☐	NON ☐	IN ☐

PERSONNES SUSPECTEES DE SOUFFRIR D'EPILEPSIE A REFERER VERS LE CENTRE DE SANTE (INFIRMIER/MEDECIN/NEUROLOGUE):

CODE UNIQUE (E/VV/MMM/PP): __/__ __/__ __ __/__ __ Nom: ________________________________ Genre (M/F): __ Age: ______ (ans/mois)
CODE UNIQUE (E/VV/MMM/PP): __/__ __/__ __ __/__ __ Nom: ________________________________ Genre (M/F): __ Age: ______ (ans/mois)
CODE UNIQUE (E/VV/MMM/PP): __/__ __/__ __ __/__ __ Nom: ________________________________ Genre (M/F): __ Age: ______ (ans/mois)
CODE UNIQUE (E/VV/MMM/PP): __/__ __/__ __ __/__ __ Nom: ________________________________ Genre (M/F): __ Age: ______ (ans/mois)
